# Impact of Obesity on the Expression Profile of Natriuretic Peptide System in a Rat Experimental Model

**DOI:** 10.1371/journal.pone.0072959

**Published:** 2013-08-29

**Authors:** Manuela Cabiati, Serena Raucci, Tiziana Liistro, Eugenia Belcastro, Tommaso Prescimone, Chiara Caselli, Marco Matteucci, Patricia Iozzo, Letizia Mattii, Daniela Giannessi, Silvia Del Ry

**Affiliations:** 1 CNR Institute of Clinical Physiology, Pisa, Italy; 2 Scuola Superiore Sant’Anna, Pisa, Italy; 3 Laboratory of Medical Science, Institute of Life Sciences, Scuola Superiore Sant’Anna, Pisa, Italy; 4 Department of Clinical and Experimental Medicine, Unit of Histology and Medical Embryology, University of Pisa, Pisa, Italy; Paris Institute of Technology for Life, Food and Environmental Sciences, France

## Abstract

Natriuretic peptides (NPs) play an important role in obesity and aim of this study was to evaluate, in cardiac tissue of obese Zucker rats (O, n = 29) their transcriptomic profile compared to controls (CO, n = 24) by Real-Time PCR study; CNP protein expression was evaluated by immunostaining and immunometric tests. Myocardial histology was performed, confirming no alteration of organ structure. While ANP and BNP are cardiac peptides, CNP is mainly an endothelial hormone; thus its expression, as well as that of NPR-B and NPR-C, was also evaluated in kidney and lung of an animal subgroup (n = 20). In heart, lower BNP mRNA levels in O vs CO (p = 0.02) as well as ANP and CNP (p = ns), were detected. NPR-B/NPR-A mRNA was similar in O and CO, while NPR-C was numerically lower (p = ns) in O than in CO. In kidney, CNP/NPR-B/NPR-C mRNA was similar in O and CO, while in lung CNP/NPR-C expression decreased and NPR-B increased (p = ns) in O vs CO. Subdividing into fasting and hyperglycemic rats, the pattern of mRNA expression for each gene analyzed remained unchanged. The trend observed in heart, kidney and lung for CNP protein concentrations and immunohistochemistry reflected the mRNA expression. TNF-α and IL-6 mRNA were measured in each tissue and no significant genotype effect was detected in any tissue. The main NP variations were observed at the cardiac level, suggesting a reduced release by cardiac cells. The understanding of mechanisms involved in the modulation of the NP system in obesity could be a useful starting point for future clinical study devoted to identifying new obesity treatment strategies.

## Introduction

Obesity is a global epidemic disease [1], [2], and ongoing investigation has shown that the number of individuals categorized as overweight or obese has increased over the last three decades and is continuing to rise, especially in industrialized countries [3]. The progression of obesity has been associated with an increased predisposition for developing additional pathological conditions, such as cardiovascular disease risk factors (e.g., diabetes, hypertension, dyslipidemia, prothrombotic and pro-inflammatory environments), respiratory dysfunction (obstructive sleep apnea), renal pathologies and a wide array of malignancies [4]–[10]. It is well-established that several risk factors for cardiovascular disease in addition to the progression of obesity lead to the development of a condition termed “metabolic syndrome”, which can dramatically worsen the clinical outcome of afflicted patients [11]–[13]. The key components of metabolic syndrome are abdominal obesity, atherogenic dyslipidemia, elevated blood pressure, glucose intolerance, and pro-inflammatory and prothrombotic states [14], [15]. The deregulation of neurohormonal systems, including the natriuretic peptide (NP) system, may increase visceral fat mass and contribute to the development of insulin resistance in dysmetabolic patients [16]. In normal subjects, NPs may affect the homoeostasis of glucose and lipid metabolism, partly through the reduction of adipogenesis [16]. The action of NPs is mainly performed at the cardiac level. They are involved in cardiovascular and endocrine homoeostasis through the regulation of body fluid and blood pressure, both by promoting diuresis and natriuresis, and by modulating vascular inflammation and cardiovascular remodeling [17]–[19]. In obesity, NPs are not new players in metabolic regulation and it was found that they are abnormally regulated [20]. They are significantly lower in overweight and obese patients compared with lean patients [21]. There are several potential mechanisms responsible for the inverse association between NP plasma levels and body mass index [22], [23]; among these are the augmented concentration of the NP clearance receptor on adipocyte cells and/or the impaired synthesis and release of NPs from myocytes in obese subjects [24]. The role of both atrial natriuretic peptide (ANP ) and B-type natriuretic peptide (BNP) during lipolysis/lipogenesis is largely defined [25], [26] while the action of C-type natriuretic peptide (CNP), the third member of the NP family, is not completely clear [27]. Recent evidence suggests that CNP is an important natural regulator of adipogenesis through binding with its specific receptor, NPR-B, and may function as an autocrine/paracrine marker in the early phase of this process [28]. In the same report, it is shown that CNP enhances adiposity through both lipid accumulation and an increase in adipogenesis marker genes. These observations suggest an important involvement of the CNP/cGMP system in the regulation of biological events that are different from those of the ANP/cGMP system; the CNP/cGMP system regulates the early stage of adipogenesis, while the ANP/cGMP system acts on mature adipocytes, controlling the activation of hormone-sensitive lipase [28].

A link between CNP and the pro-inflammatory state in obesity has been suggested. Adipose tissue releases a variety of factors, including cytokines (IL-6 and IL-1), tumor necrosis factor (TNF)-α and chemokines [29], [30] able to regulate CNP transcription and stimulate the release of this peptide, suggesting either a possible interplay between macrophage cytokine production and vascular tissue or a pivotal role of CNP in obesity and related pathologies.

To the best of our knowledge, studies on obesity carried out to evaluate the simultaneous determination of NPs and their receptors in cardiac tissue, as well as the involvement of the CNP/NPR-B system in organs that develop collateral damage, such as kidney and lung, are still lacking. The aim of our study was to evaluate the NP system profile in cardiac tissue of obese Zucker rats, in order to verify the impaired synthesis and release of these peptides during obesity. Moreover, as CNP is mainly of endothelial origin, in a subgroup of animals its transcription level alterations, as well as those of NPR-B and NPR-C, were also evaluated in kidney and lung. To assess the inflammatory profile, TNF-α and IL-6 mRNA were also measured.

## Materials and Methods

### Ethics Statement

National guidelines for the care and use of research animals (D.L. 116/92, implementation of EEC directive 609/86) were followed.

### Experimental Animal Model

Our study was carried out exploiting an eligible animal model mimicking obesity. The Zucker rat model offers a reliable genetic model for research on obesity and metabolic syndrome due to the incorporation of transgenic and knock-out technology [31], [32]. The obese Zucker rat bears a mutation in the leptin receptor gene, triggering an impaired satiety reflex that leads to obesity [11], [33]–[36].

As reported by manufacturer (Charles River Laboratories International, Inc., Wilmington, MA, USA), Zucker rats present hyperlipemia and hypertension, and exhibit severe hepatic as well as peripheral insulin resistance. The study included 53 male Zucker rats 9–13 weeks of age, subdivided into two groups: obese rats (O, n = 29, body weight = 362±8.6 g), and age-matched lean rats (CO, n = 24, body weight = 286±9.7 g) as control. Rats were fasted for 12 h with unrestricted access to water. Part of each group was studied during fasting conditions (CO*_fc_*, n = 10, glycemia = 106.8±14.3 mg/dl; O*_fc_*, n = 16, glycemia = 305.6±34.3 mg/dl) and the remainder during the induction of acute hyperglycemia (CO*_AH_*, n = 15, glycemia = 314.4±23.1 mg/dl; O*_AH_*, n = 12, glycemia = 416.9±36.8 mg/dl). Hyperglycemia was induced by injecting a 0.5-ml bolus of a 50% glucose solution.

On the day of the study, anesthesia was induced by inhalation of 2% isouorane and maintained by intra-peritoneal administration of Zoletil (Virbac, srl) (tiletamine and zolazepam 40 mg kg^−1^) and Xylazine (5 mg/kg^−1^) [37].

During the procedure, the animals were under general anesthesia and sacrificed by isouorane overdose. The tissue was collected from the left ventricle, kidney and lung and immediately placed in ice-cold RNAlater (Qiagen S.p.A, Milano, Italy) and stored at −80°C for later use. Cardiac tissues were also paraffin-embedded for histological/immunoistochemical analysis.

These procedures were notified by the Italian Ministry of Health in accordance with Italian law.

(*The name of the IACUC or ethics committee was not reported because it is not required by Italian law*).

### Tissue Handling, RNA Extraction and cDNA Synthesis

Cardiac (n = 53), renal (n = 20) and pulmonary (n = 20) tissue was homogenized with an automated tissue lyser through high-speed shaking in plastic tubes with stainless steel beads (Qiagen S.p.A, Milano, Italy).

Extraction of both protein and mRNA was performed from a single sample of each tissue. An initial extraction step using the acid guanidinium thiocyanate-phenol-chloroform method was performed and RNA was subsequently isolated from the upper aqueous phase while total proteins were obtained from the bottom organic phase and processed following manufacturer’s instructions (Rneasy Fibrous Midi kit, Qiagen GmbH, Hilden, Germany) as previously described [38]–[40].

RNA concentration and purity were determined spectrophotometrically (BioPhotometer, Eppendorf Italy, Milan, Italy), measuring spectral absorption at 260 nm. The integrity and purity of total RNA was also detected by electrophoresis of samples on Gel Star Stain (Lonza, Switzerland) agarose gels. Samples showing clear and distinct 28S and 18S ribosomal RNA bands and having spectrophotometric OD 260/280 ratios of 1.9–2.1 were used. A known amount of total RNA (Ambion Inc., USA) was used as marker. The RNA samples were stored at −80°C for use in gene expression studies.

Following DNAse treatment (RNase-Free DNase Set, Qiagen S.p.A, Milano, Italy), first strand cDNA was synthesized with an iScript cDNA Synthesis kit (Bio-Rad, Hercules, CA, USA) using about 1 µg of total RNA as template. Reverse transcriptase reaction sequence consisted of incubation at 25°C for 5 min, followed by three different cycles at 42°C for 30 min and 45°–48°C for 10 min, in order to better separate the strands. The reverse transcriptase enzyme was inactivated by heating to 85°C for 5 min. The cDNA samples obtained were placed on ice and stored at 4°C until further use.

### Protein Extraction

Tri-reagent procedure (Molecular Research Center, Cincinnati, OH, USA) allowed obtaining RNA and proteins from a single sample using a monophasic mixture of phenol and guanidine thiocyanate and isopropyl alcohol to precipitate nucleic acids, as previously described [38], [39]. Proteins isolated by organic phase were added to ethanol and centrifuged to eliminate the lipid component. Proteins were isolated by organic phase. Subsequently, the addition of acetone and centrifugation (12,000×*g*, 5 min at 4°C) made up a protein pellet that was washed and centrifuged three times with a wash buffer (guanidine, glycerol 25%, ethanol 96%). After a last wash with a solution of glycerol 25% and ethanol the pellet was re-suspended with Tris (hydroxy–methyl–aminomethane) HCl (4 mM) buffer (pH 7.4) (NaCl-154 mM, phenyl–methyl–sulfonylfluoride–PMSF-0.1 mM, sodium dodecyl sulfate (SDS) 2%). The final protein preparations were frozen at −20°C and the protein concentration was determined according to the method of Lowry using BSA as a standard [41].

### Real-Time PCR

Real-Time PCR reactions were performed in duplicate in the Bio-Rad C1000™ thermal cycler (CFX-96 Real-Time PCR detection systems, Bio-Rad Laboratories Inc., Hercules, CA, USA) as previously described [40]. For monitoring cDNA amplification a third-generation fluorophore, EvaGreen, was used (SsoFAST EvaGreen Supermix, Bio-Rad). PCR was performed in a volume of 20 µl per reaction; to minimize the influence of PCR inhibitors in Real-Time applications, all cDNA samples were diluted 1∶5. Reaction mixture included 2 µl of template cDNA [100 ng], 0.2 µM of each primer (Sigma-Aldrich, St. Louis, MO, USA), 1X SsoFAST EvaGreen SuperMix (BioRad) and sterile H_2_O. Amplification protocol started with 98°C for 30 s followed by 40 cycles at 95°C for 5 s and 60°C for 30 s. To assess product specificity, amplicons were systematically checked by melting curve analysis. Melting curves were generated from 65°C to 95°C with increments of 0.5°C/cycle. Multiple inter-run calibrators were always used to allow comparison of Ct values obtained in different runs.

The primer pairs specific for each gene analyzed in this study were designed with Primer Express (Version 2 Applied Biosystems) and reported in Table 1; whenever possible, intron-spanning primers were selected to avoid amplification of genomic DNA. Reaction conditions of all primer pairs used were optimized; i.e., a gradient PCR was conducted to assess the optimal annealing temperature while a standard curve obtained by scalar dilution of a cDNA pool (1∶5, 1∶25, 1∶125, 1∶625) was always generated to verify PCR efficiency.

**Table 1:** Details of specific primers used in Real-Time PCR experiments.

GENE	PRIMER SEQUENCE	GenBank, accession #	AMPLICON bp	Ta, °C	EFFICIENCY, %	R^2^
**Sdha**	**F:** CTCTTTTGGACCTTGTCGTCTTT	NM_130428	102	60	104.7	0.999
** 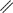 **	**R:** TCTCCAGCATTTGCCTTAATCGG	** 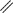 **	** 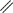 **	** 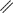 **	** 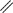 **	** 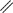 **
**Hprt1**	**F:** CCCAGCGTCGTGATTAGTGATG	NM_012583	125	60	103	0.998
** 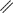 **	**R:** TTCAGTCCTGTCCATAATCAGTC					
**Tbp**	**F:** CACCGTGAATCTTGGCTGTAAAC	NM_001004198	123	60	105	0.998
** 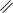 **	**R:** CGCAGTTGTTCGTGGCTCTC	** 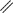 **	** 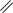 **	** 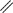 **	** 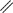 **	** 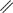 **
**ANP**	**F:** AAGTGTAGATGAGTGGTT	NM_012612	118	58	95.3	0.999
** 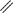 **	**R:** TGGTGCTGAAGTTTATTC	** 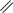 **	** 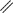 **	** 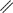 **	** 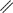 **	** 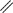 **
**BNP**	**F:** ATCTGTCGCCGCTGGGAGGT	NM_031545	187	60	101.1	0.999
** 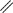 **	**R:** TGGATCCGGAAGGCGCTGTCT	** 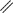 **	** 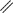 **	** 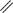 **	** 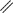 **	** 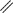 **
**CNP**	**F:** GGAGCCAATCTCAAGGGA	NM_053750	201	60	103.9	0.997
** 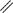 **	**R:** TGCCGCCTTTGTATTTGC	** 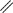 **	** 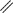 **	** 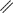 **	** 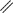 **	** 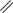 **
**NPR-A**	**F:** AGAGCCTGATAATCCTGAGTA	NM_012613	81	58	95.4	0.998
** 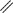 **	**R:** A TCCACGGTGAAGTTGAAC	** 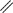 **	** 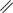 **	** 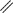 **	** 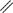 **	** 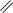 **
**NPR-B**	**F:** CCCATCCTGTGATAAAACTCC	NM_053838	89	60	104	0.999
** 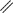 **	**R:** AAGCTGGAAACACCAAACA	** 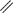 **	** 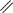 **	** 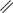 **	** 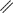 **	** 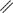 **
**NPR-C**	**F:** GGACCGCGAAGCCTGAGTTTGAGA	NM_012868	240	64	100.3	0.998
** 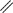 **	**R:** ATGGACACCTGCCCGGCGATACCT	** 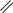 **	** 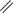 **	** 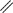 **	** 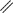 **	** 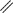 **
**IL-6**	**F:** ACCACCCACAACAGACCAGT	NM_012589	141	60	96	0.997
** 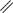 **	**R:** ACAGTGCATCATTCGCTGTTC	** 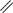 **	** 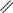 **	** 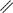 **	** 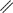 **	** 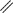 **
**TNF-α**	**F:** GCCCAGACCCTCACACTC	NM_012675	98	60	105	0.997
** 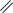 **	**R:** CCACTCCAGCTGCTCCTCT	** 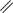 **	** 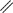 **	** 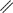 **	** 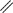 **	** 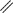 **

**Legend: Sdha:** Succinate dehydrogenase complex, subunit A, flavoprotein; **Hprt1:** Hypoxanthine phosphoribosyltransferase 1; **Tbp:** TATA binding protein; **ANP:** atrial natriuretic peptide; **BNP:** B-type (or brain) natriuretic peptide; **CNP:** C-type natriuretic peptide; **NPR-A:** Natriuretic peptide receptor A; **NPR-B:** Natriuretic peptide receptor B; **NPR-C:** Natriuretic peptide receptor C or clearance receptor; **IL-6:** Interleukin-6; **TNF-α:** Tumor necrosis factor-alpha.

### CNP Assay

CNP was directly measured in cardiac (n = 20), renal (n = 20) and pulmonary (n = 20) protein extracts by a specific radioimmunoassay (C-type peptide-22 [CNP-22] human, porcine, rat magnetic bead RIA KIT, Phoenix Pharmaceuticals, Belmont, CA, USA). Each sample was assayed in duplicate and the experiment was carried out in an ice bath. A control sample, prepared using known amounts of CNP standard added to the assay buffer, was assayed in each run for quality control.

### Histological and Immunohistochemical Analysis

Five µm-thick sections of cardiac tissue of CO and O were deparaffined, rehydrated and processed for both routine haematoxylin-eosin staining and immunohistochemistry. Immunohistochemistry, used to evaluate CNP expression, was carried out as previously described [42]. Briefly, after antigen retrieval and endogenous peroxidases/non specific binding blocking, the samples were incubated with a rabbit anti-CNP antibody (CNP-53, Phoenix Pharmaceuticals, Inc., CA, USA) diluted 1∶800 in 1% bovine serum albumin/phosphate buffered saline (BSA/PBS). Detection was performed by sequential treatments with biotinylated anti-rabbit immunoglobulins, streptavidin-peroxidase complex and DAB (Vector, Burlingame;CA, USA). For each sample, at least 3 sections were examined by two independent observers using a semi-quantitative scale of immunoreactivity, consisting of no (−), low (+) and high (++) staining. Photomicrographs were taken using a DFC480 digital camera (Leica Microsystem, Cambridge, UK). Negative controls were obtained by incubating specimens in the absence of the primary antibody.

### Data Analysis

#### Statistical analysis

The geometric mean of the three most stably expressed genes in each specific tissue (heart, kidney and lung, respectively) settled in a previous work [40], was used for normalization of Real-time PCR results.

Relative quantification of each target gene studied was calculated by the ΔΔCt method.

All sample values and other data for quality control of the RIA system were calculated by a previously described computer program; interpolation of the dose-response curves was computed using a four-parameter logistic function [43].

Group comparison was performed by Student’s t-test or analysis of variance (ANOVA) as appropriate, using Statview 5.0.1 software released for Windows Statistical (SAS Institute, Inc., Cary, NC, USA).

Relations between variables were assessed by linear regression.

The results are expressed as mean ± SEM and *p-value* was considered significant when <0.05.

## Results

Body weights, baseline glucose and insulin levels were evaluated with conventional methods in both obese Zucker rats and control lean animals, documenting that obese Zucker rats were 20% heavier than the CO group (O: 362±8.6 vs CO: 286±9.7 g, p<0.0001) with baseline glycemia values of O: 351.7±26.8 vs CO: 236.5±25.9 mg/dl, p = 0.004 and a baseline insulin of O: 2633±450 vs CO: 1143±224 pg/ml, p = 0.009.

### Assessment of Real-Time PCR Condition

To optimize the thermocycle profile of each reference gene, optimal annealing temperature and RNA concentration was assessed for each designed PCR primer.

Dilution series were run for all candidate reference genes to quantify Real-time PCR efficiency that resulted in the range of 95–105% and a linear standard curve, R^2^, greater than |0.990|(Table 1).

### Gene Expression Profiling

All the target genes analyzed were normalized with the three most stably expressed genes in the left ventricle (Sdha, Tbp, Hprt1), kidney (Actb, Gapdh, Tbp) and lung (Hprt1, Ywhag, Sdha), as previously described [40].

**Figure 1:**
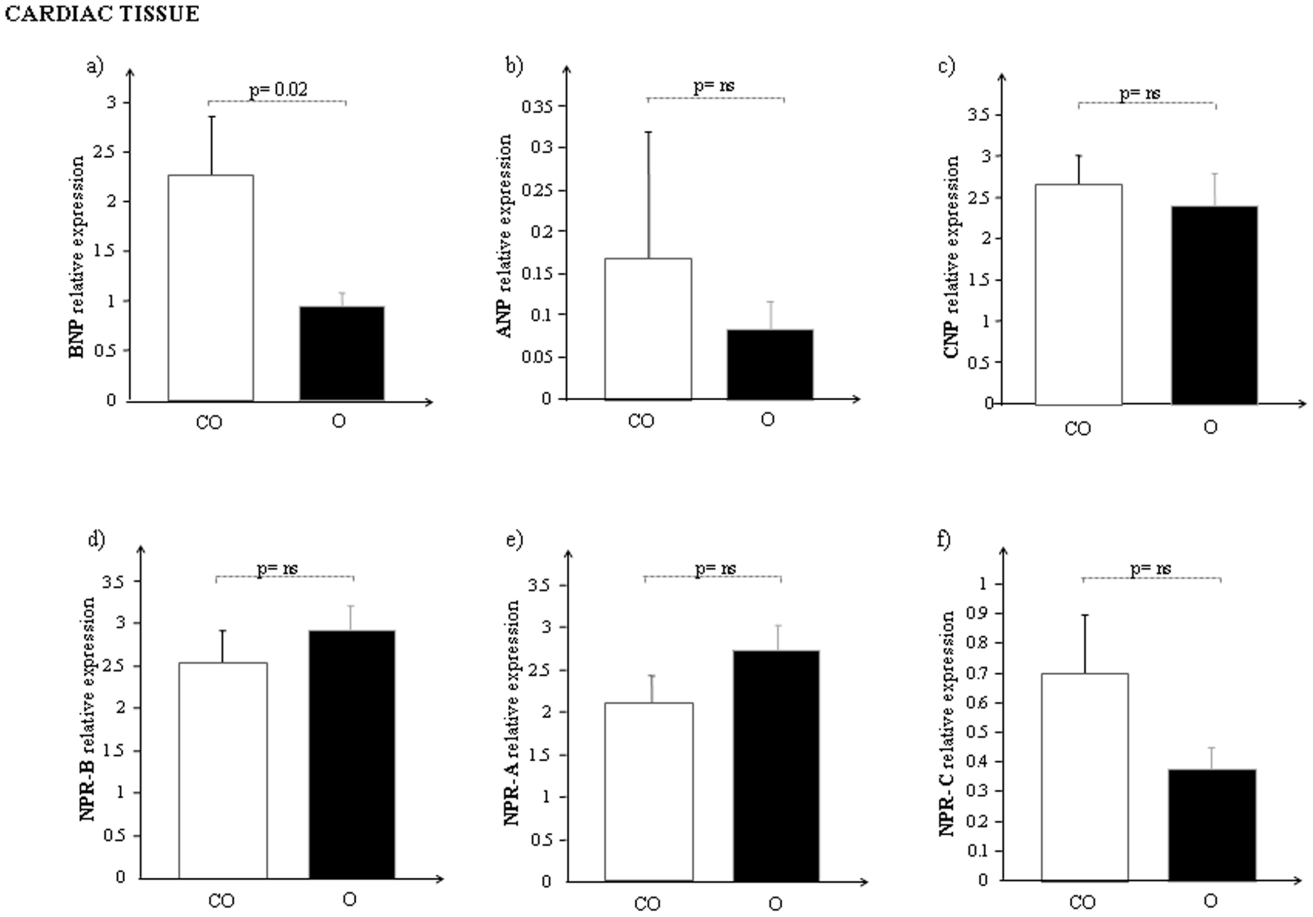
mRNA expression of a) BNP, b) ANP, c) CNP, d) NPR-B, e) NPR-A and f) NPR-C in cardiac tissue of control lean (CO, white bar) and obese Zucker rats (O, black bar).

The global changes in mRNA expression of the NP system in the heart of both obese and lean control Zucker rats are reported in Fig. 1.

BNP mRNA expression levels resulted significantly lower (Fig. 1a, p = 0.02) in cardiac tissue of obese rats with respect to controls while ANP (Fig. 1b) and CNP (Fig. 1c) showed a reduction of mRNA expression but it was not significant.

Regarding the NP receptor system any statistical difference was not observed (Fig. 1 d–f): the mRNA cardiac expression of both NPR-B and NPR-A resulted slightly higher in O than in CO, while the clearance receptor, NPR-C resulted down-regulated in O compared to CO.

**Figure 2:**
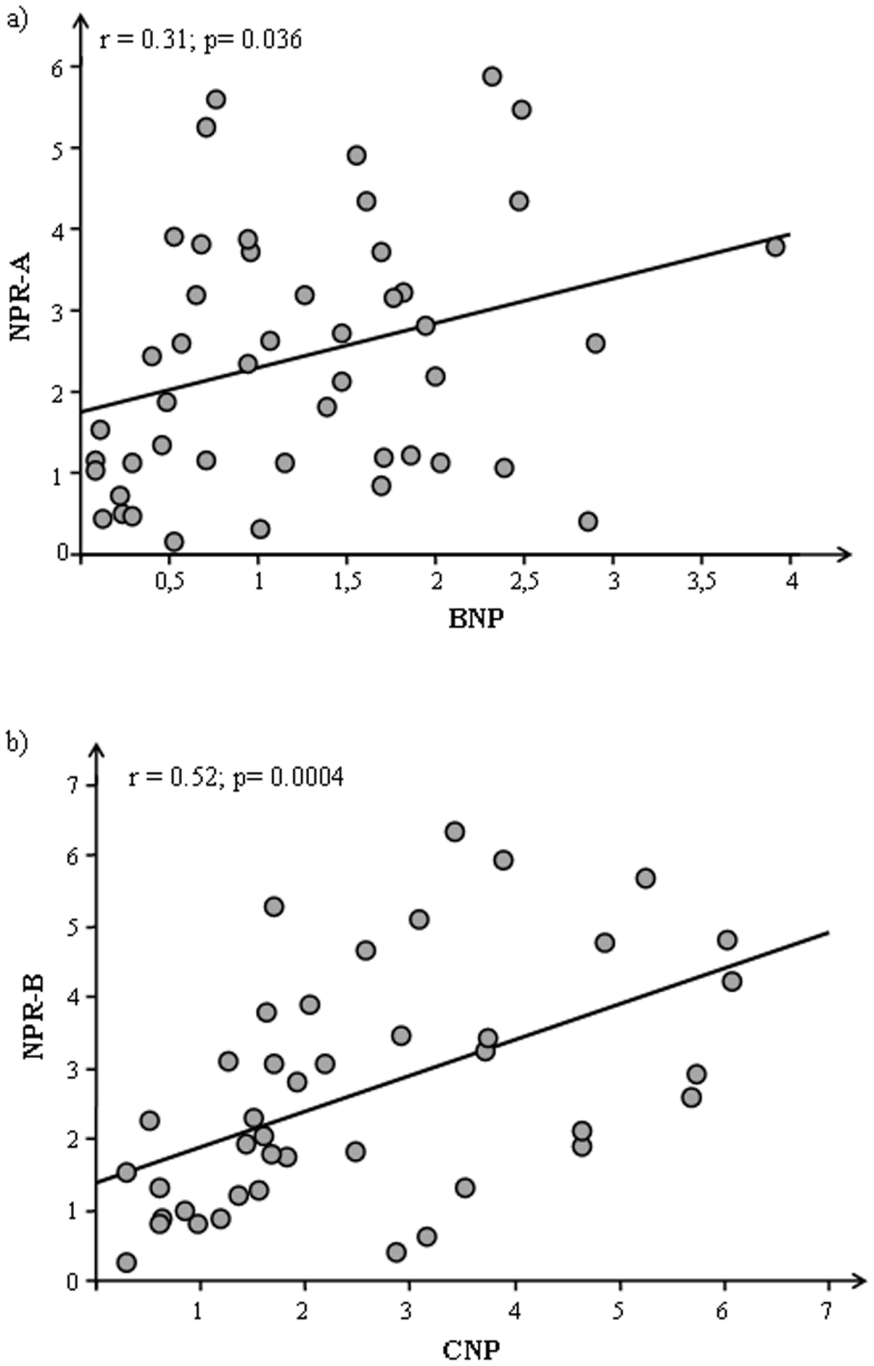
Correlations between a) BNP and NPR-A b) CNP and NPR-B in cardiac tissue of Zucker rats (CO+O).

Considering the animals as a whole significantly correlations were observed for BNP and CNP with their specific receptors NPR-A (p = 0.036) and NPR-B (p = 0.0004), respectively (Fig. 2) but splitting by each genotype the significance remains only for obese group (BNP-NPR-A: p = 0.0024; CNP-NPR-B: 0.0024).

**Figure 3:**
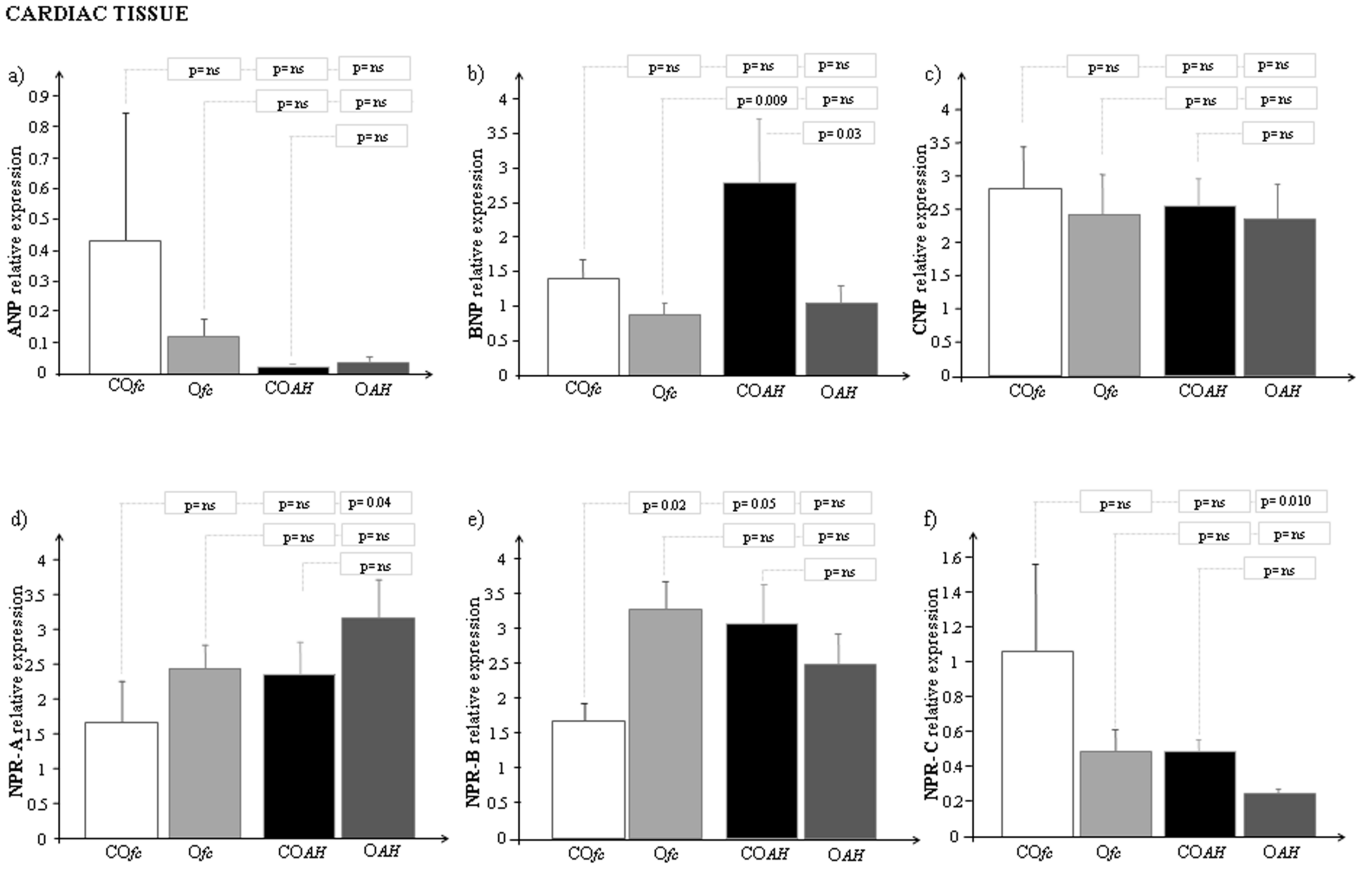
mRNA expression of a) ANP, b) BNP, c) CNP, d) NPR-A, e) NPR-B and f) NPR-C in cardiac tissue of control lean and obese Zucker rats (during fasting, and during acute hyperglycemia). *[GRAPH LEGEND: CO_fc_: Control lean Zucker rats during fasting conditions (white bar); O_fc_: Obese Zucker rats during fasting conditions (light grey bar); CO_AH_: Control lean Zucker rats during acute hyperglycemia (black bar); O_AH_: Obese Zucker rats during acute hyperglycemia (grey bar)].*

When the two groups, lean and obese Zucker rats, were further divided and studied during fasting conditions (CO*_fc_* and O*_fc_*) and during the induction of acute hyperglycemia (CO*_AH_* and O*_AH_*), significant mRNA expression levels were obtained for BNP in the hyperglycemic group (CO*_AH_* vs O*_AH_,* p = 0.03) and for NPR-B in fasting rats (CO*_fc_* vs O*_fc_,* p = 0.02) (Fig. 3).

There are some interesting findings to note about the expression of natriuretic peptides after induction of hyperglycemia showing specific response for each natriuretic peptide in control rats: down and up-regulation for ANP and BNP respectively and no response for CNP (Fig. 3a–c). Moreover, in the same samples a different regulation was also observed for the natriuretic peptide receptors: up-regulation for NPR-A and NPR-B with a down-regulation for NPR-C (Fig. 3d–f).

**Figure 4:**
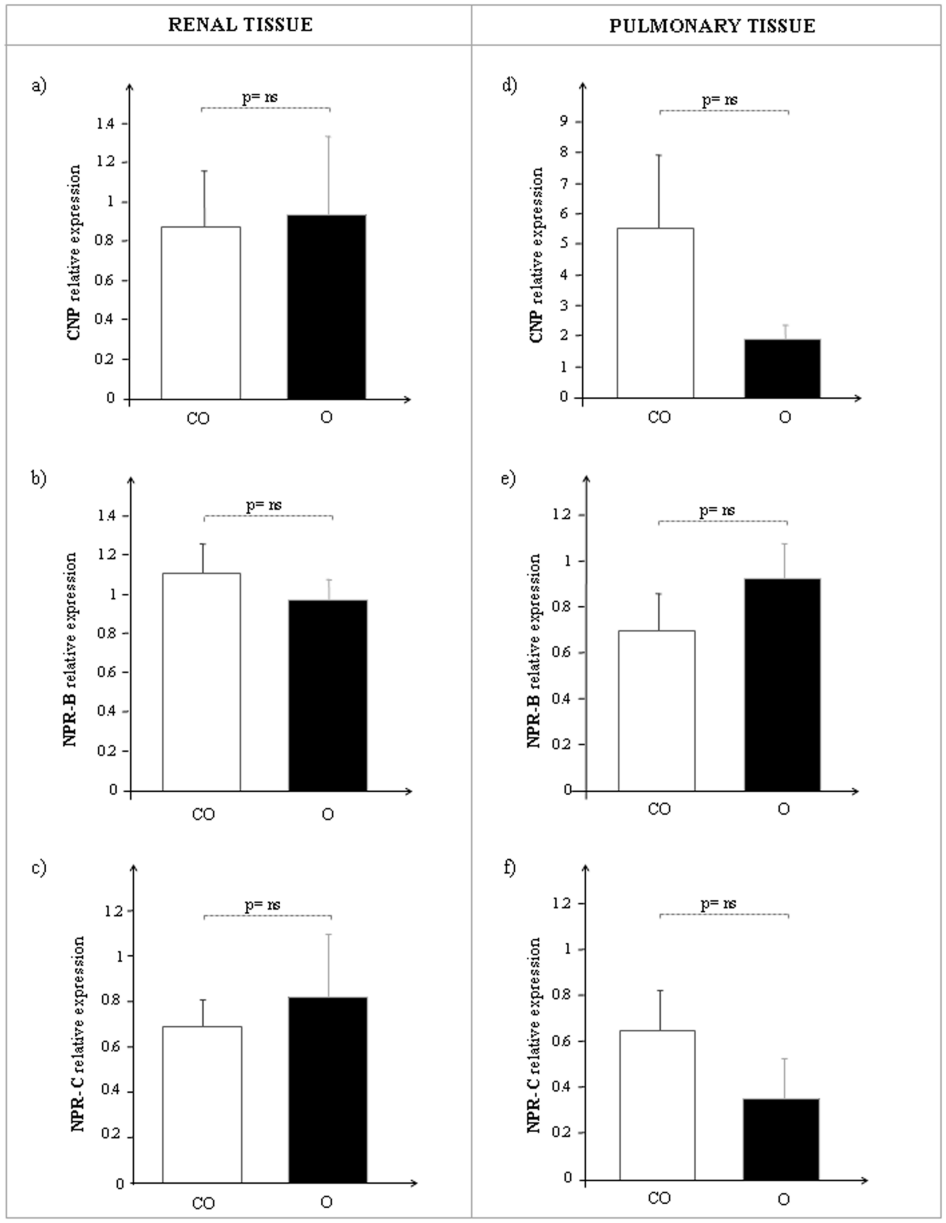
CNP, NPR-B and NPR-C mRNA expression in renal and pulmonary tissue of control lean (CO, white bar) and obese Zucker rats (O, black bar). On the left side: **a)** CNP, **b)** NPR-B, **c)** NPR-C relative levels in kidney. On the right side: **d)** CNP, **e)** NPR-B, **f)** NPR-C relative levels in lung.

In the kidney, CNP, NPR-B and NPR-C mRNA expression was similar in both O and CO rats (Fig. 4 a–c), while in the lung CNP and NPR-C transcription levels decreased in parallel with an increased NPR-B expression, although not significantly, in obese Zucker rats with respect to controls (Fig. 4 d–f). Subdividing into fasting and hyperglycemic rats, the pattern of mRNA expression for each gene analyzed remained unchanged.

### CNP Immunometric Determination in Cardiac, Renal and Pulmonary Tissue

CNP extract tissue levels were measured in heart, kidney and lung of CO and O.

The pattern observed at the mRNA level was confirmed by CNP protein concentration in heart (CO: 0.36±0.07 vs O: 0.26±0.07, p = ns), kidney (CO: 0.20±0.03 vs O: 0.16±0.01, p = ns) and lung (CO: 0.46±0.03 vs O: 0.41±0.02, p = ns).

### Inflammatory Profile in Cardiac, Renal and Pulmonary Tissue of TNF-α and IL-6

**Figure 5:**
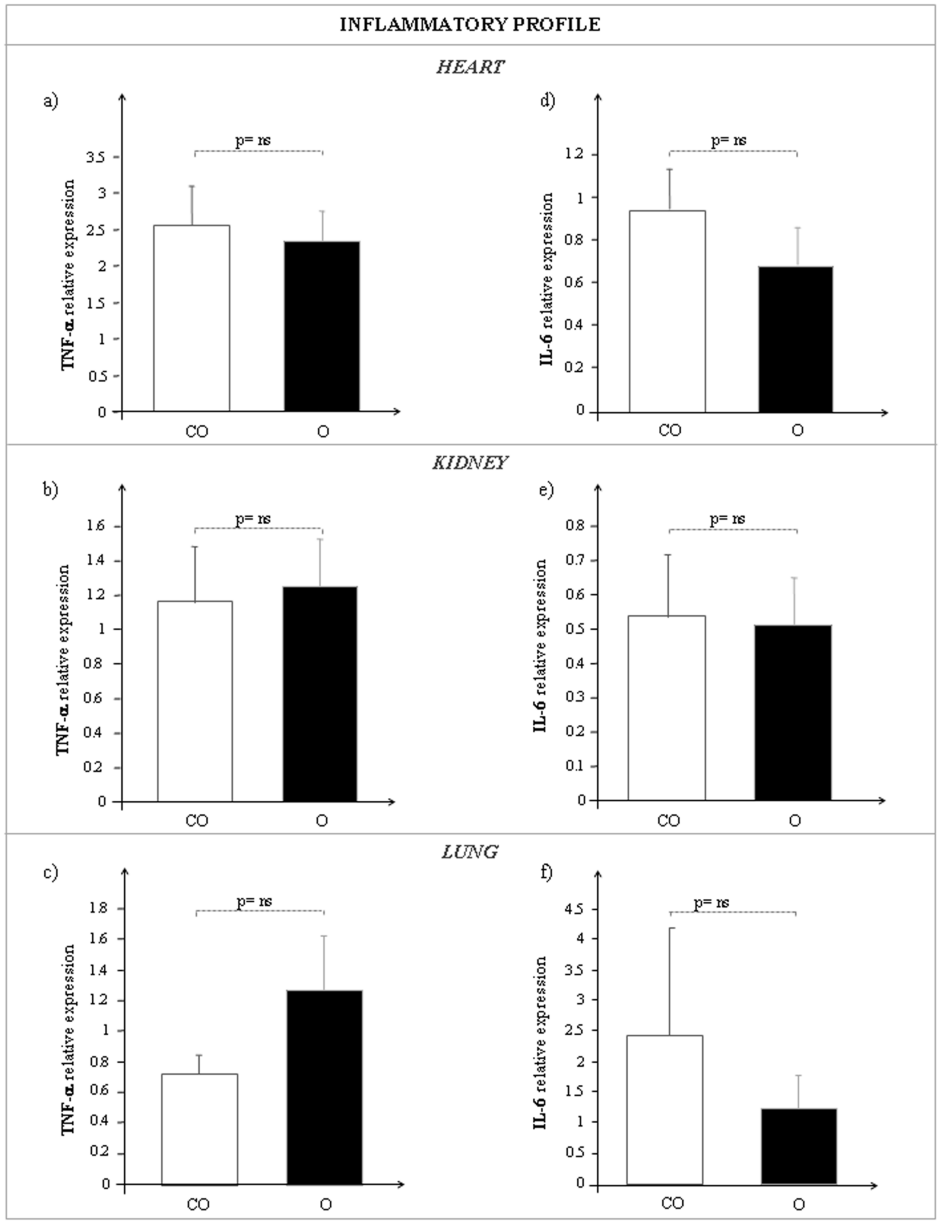
TNF-α (a, b, c) and IL-6 (d, e, f) mRNA expression in heart (upper side), kidney (middle side) and lung (bottom side) of control lean (CO, white bar) and obese Zucker rats (O, black bar).

**Figure 6:**
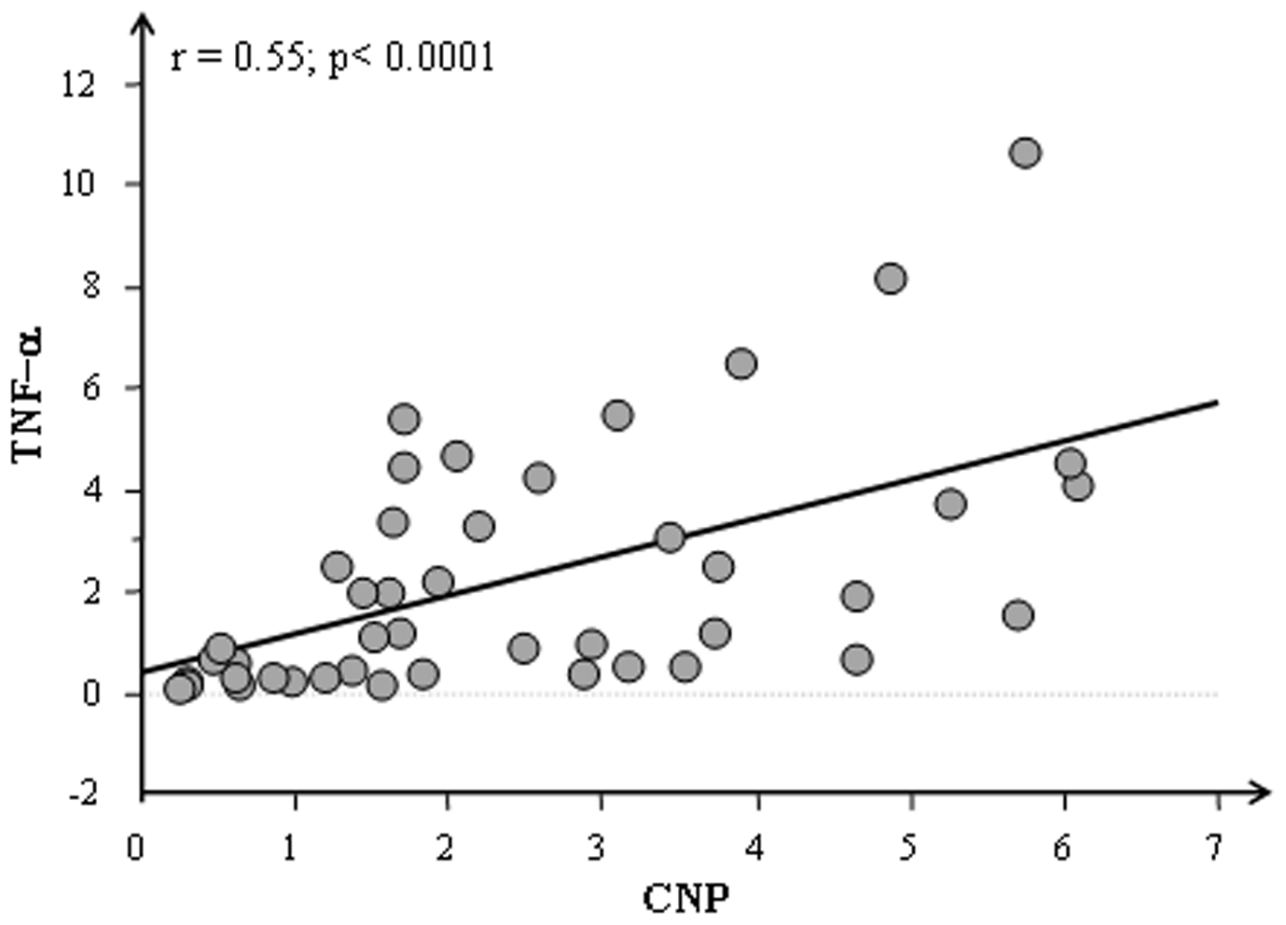
Correlations between CNP and TNF-α in cardiac tissue of Zucker rats (CO+O).

The mean expression values obtained for TNF-α and IL-6 in heart, in kidney and in lung of lean and obese Zucker rats are reported in Fig. 5. Similar expression pattern for both TNF-α and IL-6 was observed in cardiac, renal and pulmonary tissues of CO and O rats. A highly significant correlation between CNP and TNF-α (p<0.0001) was detected in cardiac tissue (Fig. 6).

### Myocardial Structure

**Figure 7:**
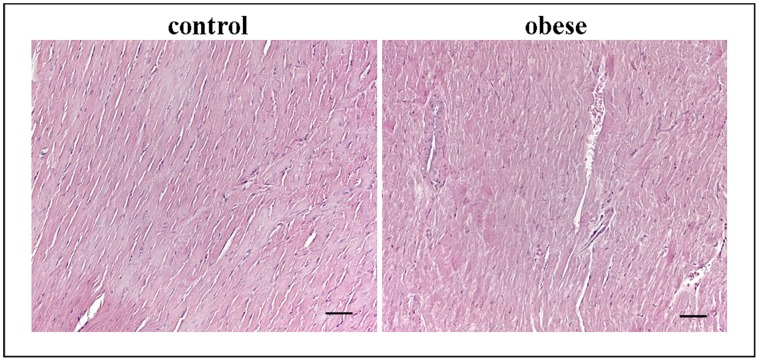
Representative hematoxylin/eosin staining from heart sections of controland obese rats. **Scale bar: 50 µm.**

Histopathologic analysis, carried out by haematoxylin and eosin staining, revealed in all heart samples, obese and controls, a normal morphology without alterations of myocardial structure as the disruption of cardiac fibers, hemorrhages or leukocyte infiltrate (Fig. 7).

### Myocardial CNP Immunohistochemistry

**Figure 8:**
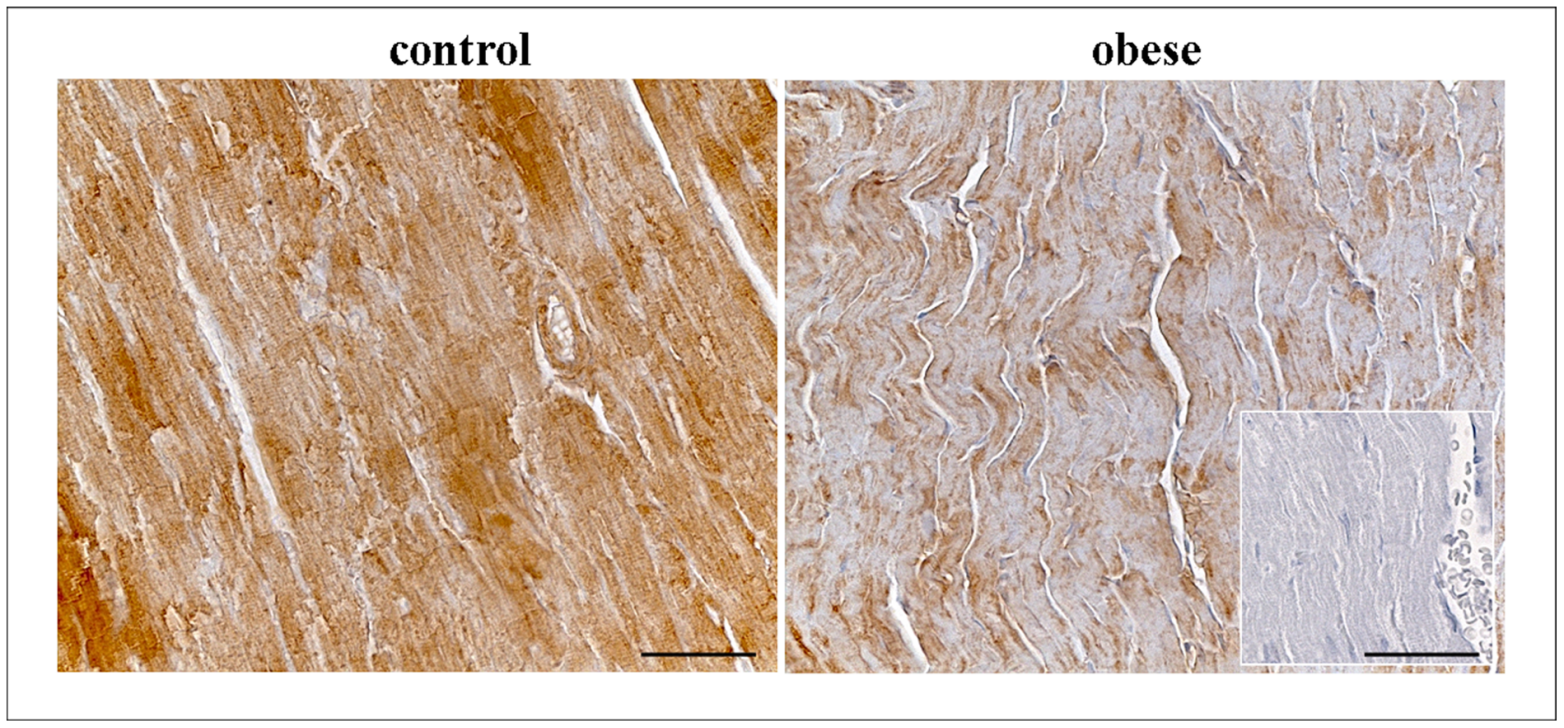
Immunohistochemical staining of CNP in the cardiac tissue of control and obese rats. **Negative control was showed in the square. Scale bar: 50 µm.**

Myocardial CNP expression was assessed by immunohistochemistry to confirm its transcriptional trend. As showed in Fig. 8 CNP immunoreaction (brown) was greater in CO (+++) than O (+) while the negative control was not immunoreactive (−).

## Discussion

The data obtained in our paper have evidenced significantly lower levels of BNP mRNA and a reduction of ANP and CNP transcript levels in cardiac tissue of obese rats with respect to controls, confirming previous observations about the role of the NP system in obesity [21], .

Unlike other natriuretic peptides, BNP significantly decreases in obese rats but the different behavior of each peptide can be justified by their different secretion sources; in fact, while ANP and BNP are primarily cardiac hormones secreted mainly from the atria and ventricles respectively, CNP is released mainly from endothelial cells and to a lesser extent from cardiomyocytes [42]. CNP is widely expressed throughout the vasculature and is found in particularly high concentrations in the endothelium [46]–[48] where it is able to induce vasorelaxation by hyperpolarization. Thus, while ANP and BNP are typically cardiac hormones, CNP can exert its functions in other organs rich in endothelial cells. For this its expression, together with NPR-B and NPR-C mRNA, was also assessed in kidney and lung where no significant difference was found. Regarding CNP cardiac expression, probably the endothelial component greatly affects the final response. Nevertheless immunohistochemical analysis confirm a lower CNP staining in cardiac tissue of obese rats with respect to controls.

It is well-known that circulating levels of NPs are significantly lower in overweight and obese patients compared with lean subjects [24] but to date, it is not clear whether this inverse association between NP plasma levels is due to increased concentration of the NP clearance receptor on adipocyte cells [22], [23], or to an impaired synthesis and release of NPs from cardiomyocytes [24]. Our results suggest a reduced release of these peptides, especially BNP and ANP, from cardiac cells rather than the clearance action of NPR-C. However it is necessary to take into account that the lack of changes in NPR-C observed in these tissues may be due to the use of samples obtained from the homogenization of whole organs rather than from adipocytes harvested from heart, kidney and lung.

The correlation between BNP and NPR-A as well as CNP and NPR-B confirmed the different involvement of BNP and CNP in lipolysis and adipogenesis, respectively. As a matter of fact, it was recently shown that the NP-cyclic guanosine monophosphate (cGMP) signaling system appears to increase the capacity for fat oxidation, to activate mitochondrial biogenesis, and to prevent the deleterious effects of a high-fat diet [44]. Miyashita and colleagues [44] have shown an involvement of both ANP and BNP in energy metabolism under conditions of normal diet and fitness training levels compared to high-fat diet and inactivity. The results showed that ANP and BNP induced cGMP signaling through the specific biological receptor, NPR-A, whereas NPR-C inactivates these same peptides. The signaling produced by the balance of NPR-A/NPR-C promotes lipolysis [44], [49]. Katafuchi et al. showed instead that CNP is an important natural regulator of adipogenesis through binding with its specific receptor, NPR-B [28].

Histological analysis did not detect alteration of myocardial structure, confirming that NP alteration is mainly related to obesity and not to cardiac dysfunction.

When our animals were subdivided into fasting and hyperglycemic rats, the NP trend did not change. It has been recognized that a defective NP system, due to the reduced plasma levels of bioactive NPs in obese subjects, may contribute to increased visceral fat accumulation, maintaining obesity and generating insulin resistance. An impaired production, clearance and function of the NP system may lead to the development of related cardiovascular pathologies in patients suffering from metabolic alteration [50]. Moreover, evidence showed that high glucose concentration reduces NP receptor response, decreasing NP plasma levels [51], [52]. Accordingly, we found lower mRNA values of BNP in hyperglycemic obese Zucker rats without any receptor modifications in the same group.

In control rats subdivided in fasting and hyperglycemic an opposite regulation was observed for ANP and BNP mRNA expression, in line with previous reports [53], [54]. Nunes et al have reported that BNP gene expression was significantly higher in animals under sucrose treatment, in agreement with the left ventricular (LV) hypertrophy. In our study the glucose administration induces a significant variation of natriuretic peptides mRNA expression in control rats and no modification in obese rats. This discrepancy could be due to a different genotype or phenotype of the two groups. This is in line with the hypothesis that the alteration of natriuretic peptide in obese subjects may not be a consequence of increased body weight or excess adiposity, but rather may be a causative factor in the genotype or phenotype that leads to development the obesity [55].

As to ANP, the lower mRNA levels in CO*_AH_* with respect to *CO_fc_* can be justified by a balance between the synthesis and secretion. Mifune et al have observed a more intense ANP atrial immunoreattivity in hyperglycemic rats with respect to controls in parallel with a decrease of mRNA expression. Moreover, our study revealed a significant increased expression of NPR-B and a significant reduction of NPR-C in CO*_AH_* with respect to *CO_fc_* without important modifications in obese group confirming the different regulation of natriuretic peptide system in obesity.

As it was recently suggested that some forms of obesity are associated with chronic low-grade inflammation [56], this study takes into account the inflammatory process in order to obtain a more complete overview on obesity. Adipokines, cytokines secreted by adipose tissue, are released in excess during obesity and can create an environment susceptible to inflammation. In obese patients the adipose tissue undergoes molecular and cellular alterations that subsequently affect systemic metabolism; first of all, macrophages accumulate within adipose tissue, leading to systemic inflammation in both vascular and non-vascular tissues. These activated macrophages, along with adipocytes, produce several pro-inflammatory cytokines such as TNF-α and IL-6, resulting in numerous metabolic dysfunctions that accompany obesity, e.g., systemic inflammation and atherosclerosis [57].

The results presented in our study showed a correlation between TNF-α and CNP underlying a balance between inflammatory and anti-inflammatory activity.

A regular trend of activation for IL-6 mRNA levels was found in heart, kidney and lung, where its levels were almost equal or higher in obese with respect to lean Zucker rats. Anyway, the role of IL-6 in metabolic changes associated with obesity is still unclear, despite the anti-inflammatory effect suggested for IL-6 in a recent study [58].

In recent years, an attractive hypothesis has emerged proposing that those cytokines such as TNF-α and IL-6 produced by adipose tissue may be responsible for insulin resistance in obesity [59]; indeed, the expression or production of these cytokines was shown to be directly related to the degree of obesity of the subjects, and thus might be involved in obesity-related insulin resistance [59]–[63].

However, inflammation and obesity is a winding road, with most signaling molecules modifying the actions of other signaling molecules, which in turn propagate the process. For this reason, inflammation can never be merely a risk factor or an outcome of obesity; rather, it is a multifactorial contributor to the overall pathophysiology of the condition.

In conclusion, based on the above results, an alteration of the NP system in obesity was found, mainly at cardiac tissue levels compared to renal and pulmonary tissues.

The understanding of mechanisms involved in the modulation of the NP system in obesity could be a useful starting point for future clinical study devoted to identifying new obesity treatment strategies.

In this regard, the availability of novel drugs that promote the action of NPs may be an appealing therapeutic application in obesity, with particular focus on the field of cardiovascular disease.
